# Improving the Annotation for Spatial Proteomics: A Computational Approach to Enhance Molecular Characterization of Thyroid Nodules

**DOI:** 10.1021/acs.jproteome.5c00432

**Published:** 2026-01-08

**Authors:** Vasco Coelho, Nicole Monza, Natalia S. Porto, Giulia Capitoli, Vincenzo L’Imperio, Daniele M. Papetti, Vanna Denti

**Affiliations:** † Department of Informatics, Systems and Communication, 9305 University of Milano-Bicocca, 20126 Milan, Italy; ‡ Proteomics and Metabolomics Unit, Department of Medicine and Surgery, 9305 University of Milano-Bicocca, 20854 Monza, Italy; § Bicocca Bioinformatics Biostatistics and Bioimaging B4 Center, 9305 University of Milano-Bicocca, 20854 Monza, Italy; ∥ Biostatistics and Clinical Epidemiology, 9265 Fondazione IRCCS San Gerardo dei Tintori, 20900 Monza, Italy; ⊥ Department of Medicine and Surgery, Pathology, University of Milano-Bicocca, Fondazione IRCCS San Gerardo dei Tintori, 20900 Monza, Italy

**Keywords:** tissue microarray, spatial omics, proteomics, digital pathology, thyroid cancer, mass spectrometry imaging

## Abstract

The present work proposes a reproducible and automated workflow for integrating digital pathology in matrix-assisted laser-desorption ionization mass spectrometry imaging (MALDI-MSI) data analysis, using thyroid tissue as a proof-of-concept application. MALDI-MSI has shown promise in the molecular characterization of thyroid neoplasms. Yet challenges remain in minimizing signal interferents and improving diagnostic discrimination. In this study, we propose an interdisciplinary approach integrating digital pathology with spatial proteomics to enhance MALDI-MSI analysis of thyroid lesions from formalin-fixed paraffin-embedded tissue sections. We trained a pixel classifier to automatically select cell-rich regions of interest (ROIs) from hematoxylin and eosin-stained tissue microarrays, reducing interference from colloid-rich areas. The proteomics signals obtained with the pixel classifier (*PC*) were compared with those obtained from the full core (*FC*) and those manually annotated by the pathologist (*PAT*). The results showed that *PC* ROIs significantly decreased interfering signals (15%) while increasing the signal-to-noise ratio of tryptic peptides (+37%). Indeed, we detected a greater number of *m*/*z* signals (between 9 and 24%), improving the spectral clustering by means of principal component analysis to distinguish different histopathological regions. Receiver operating characteristic (ROC) analysis further confirmed the improved classification power, with a 50% increase in discriminatory *m*/*z* features across different thyroid nodules diagnosis compared to conventional *FC* and *PAT* data. Using a *PC* to select cell-specific regions globally enhances reproducibility, reduces operator workload, and optimizes MALDI-MSI workflows. Altogether, the proposed approach paves the way for more accurate molecular characterization of thyroid neoplasms and holds potential for improving biomarker discovery and diagnostic precision in clinical pathology.

## Graphical abstract

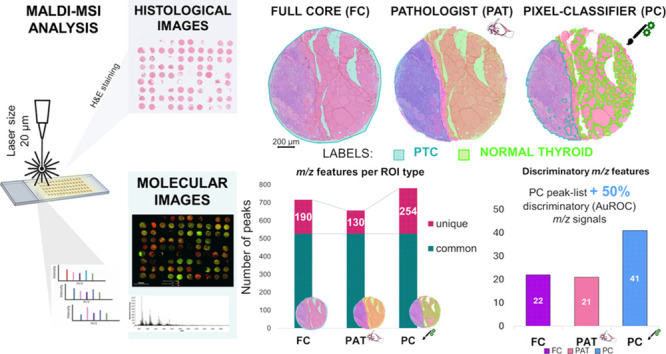

## Introduction

Among the spatial-omics techniques, matrix-assisted laser desorption/ionization-mass spectrometry imaging (MALDI-MSI) has already shown promising results in the characterization and classification of different tissue lesions.^1^ The high sensitivity of this technique allows for the measurement of relative intensity and spatial distribution of hundreds of analytes from a single tissue section.^2,3^ Additionally, MALDI-MSI permits the spatial measurement of different analyte classes (i.e., small molecules, lipids, peptides, and N-Glycans) both in fresh-frozen and archival tissue sections.^4–7^ When considering MALDI-MSI in clinical investigations, our team has extensively applied this technique for the investigation of molecular changes in thyroid neoplasms.^8–10^ For instance, MALDI-MSI proteomics analysis showed the capability to distinguish putative biomarkers that distinguish RAS-mutated tumors from wild-type tumors.^11^ In particular, the differential diagnosis of follicular-patterned thyroid lesions can be complicated due to the well-known tissue heterogeneity that comprises entities with different clinical courses and prognosis.^12,13^ Although computational pathology assists in the morphological evaluation of thyroid lesions,^14,15^ spatial proteomics approaches might improve the correct diagnosis by encoding molecular information. The possibility to perform the proteomics analysis after in situ digestion of formalin-fixed paraffin-embedded (FFPE) tissue allows the analysis of several patients at the same time using tissue microarrays (TMA).^16,17^ In this work, as a proof-of-concept, we considered four different thyroid lesions: papillary thyroid cancer (PTC), follicular-variant of PTC (FVPTC), noninvasive follicular thyroid neoplasm with papillary-like nuclear features (NIFTP), and follicular adenoma (FA). The latter represents a benign condition, whereas PTC, FVPTC, and NIFTP are malignant lesions, with the last having indolent behavior with an extremely low risk of recurrence and distant spread.^13^ However, previous studies have shown that colloid-rich regions – a gel-like substance within thyroid follicles primarily composed of thyroglobulin – in thyroid tissue can interfere with paraffin removal and consequent in situ tissue digestion, leading to signal suppression of cellular molecules and to contamination of MALDI-matrix adducts.^18^ In our case study, we observed a strong interference from α-Cyano-4-hydroxycinnamic acid (HCCA), in particular the [M4–3H+3Na+K]+ adduct (861.07 *m*/*z*),^19^ as shown in Figure S1. The direct consequence is impaired molecular characterization of thyroid nodules. A possible solution requires the manual definition of regions of interest (ROIs) that exclude colloid regions. However, this detailed work is time-consuming, suffers from interoperator variability, and is not reproducible. Therefore, automating fine-grained detection of cell-rich regions, avoiding colloid, would greatly improve the entire workflow, thus facilitating the extraction of specific proteomic signatures for each thyroid neoplasm. To do so, we trained a QuPath pixel classifier (*PC*)^20,21^ to pinpoint specific ROIs in hematoxylin and eosin (H&E)-stained thyroid tissue sections after MSI data acquisition, enabling a virtual microdissection of those areas.^3,22^ To confirm the enhanced proteomic data specificity of the *PC*-derived ROIs, we compared them with those resulting from ROIs manually annotated by an experienced pathologist, as described in the H&E-derived histopathologic ROIs subsection.

In the vision of translating MALDI-MSI to clinical routine, we used ROI annotations defined by the pathologist and not specifically designed for MSI applications. These annotations were used in our automatic pipeline in QuPath to assign labels to the PC cell-rich regions.

Moreover, our findings proved the enhanced discriminatory capability of *PC*-derived proteomic signatures in differentiating among distinct thyroid nodule diagnoses.

In the Data section, we present the bimodal spatially resolved microscopic biomedical data processed in this work: MALDI-MSI and H&E-stained whole-slide image (WSI). The proposed enhanced molecular characterization workflow is detailed in the Methods section and includes data normalization, coregistration, and accurate MALDI-MSI region selection. Moreover, in the Results and Discussion section, we applied peak-picking separately to the *PC*-derived ROIs, pathologist-guided ROIs, and full-core ROIs, and we compared the resulting peak lists based on their discriminatory capability to distinguish the labeled regions annotated by an experienced pathologist. Finally, in the Conclusions section, we discuss the advantages and flexibility of the proposed workflow for enhancing spatial omics MSI analysis and future developments for morphomolecular biomarker discovery.

## Data

The study cohort under investigation included 44 patients who underwent thyroid surgery for different thyroid lesions at the IRCCS Fondazione San Gerardo in Monza, Italy. The study was conducted in accordance with the Declaration of Helsinki and approved by the local ethical committee (Comitato Etico Brianza, via Pergolesi, 33, 20900 Monza (MB). Approval code: FINAL-TIR PU 3581/21. Approval date: January 14, 2021. All participants gave their informed consent. Two different areas of each lesion section were selected from each FFPE tissue block to build a TMA using the semiautomatic arrayer ISE Galileo TMA CK 4500 with the software ISE Galileo TMA R4.30 (Integrated Systems Engineering, Milan, Italy). The study was approved by the local ethical committee (FINAL-TIR PU 3581/21).

### MALDI–MSI Data

A 5 μm-thick section obtained from the TMA was processed for the in situ analysis of tryptic peptides, as previously described.^11^ The analysis was performed using a timsTOF fleX mass spectrometer (Bruker Daltonics, Bremen, Germany) equipped with a SmartbeamTM 3D laser, measuring in the *m*/*z* range of 700–3000. The spatial acquisition was performed using a raster width of 20 × 20 μm (*x*, *y*) and a lateral laser scan configuration of 16 μm. After the MALDI–MSI analysis, the matrix was washed with increasing concentrations of ethanol, and the slide was stained with hematoxylin and eosin (H&E). The H&E-stained slide was digitized as a WSI using a MIDI II digital scanner (3DHISTECH, Budapest, Hungary). The ROIs of each TMA core in the H&E image were manually annotated by an experienced pathologist (V.L.). The histopathologic ROIs labels comprised: PTC, FVPTC, NIFTP, FA, Hürthle cell adenoma, stroma, and normal thyroid tissue. Only samples labeled as PTC, FVPTC, NIFTP, and FA were considered in this study, leading to a total of 39 patients and 64 cores, with a maximum of 2 cores per patient.

## Methods

### MALDI–MSI Data Normalization and H&E coregistration

MALDI-MSI raw data files of each TMA core were imported into SCiLS Lab 2025a Pro software (Bruker, Bremen, Germany). Root mean square normalization was applied to the entire dataset, and patient IDs and diagnoses were assigned as attributes. The corresponding H&E-stained WSI (referred to as H&E in the rest of the manuscript for brevity) was imported and manually coregistered with the MSI data in SCiLS, a paramount step for a precise ROI selection. To improve the precision of structural alignment, coregistration was adjusted following the MSI distribution of the ion *m*/*z* 1459.688, putatively corresponding to the hydroxylated tryptic peptide (GSAGPPGATGFPGAAGR) of Collagen alpha-1­(I) chain precursor,^23^ a spatial reference for fine-structure alignment.

### H&E-Based Automatic Cell-Rich ROIs Detection

In Figure 1, we provide an overview of the workflow proposed in this work. To train the *PC* in QuPath, we manually annotated the H&E by highlighting small regions of different TMA cores, which correspond either to cell-rich regions or non-cell-rich regions. Additional annotations were iteratively added until the segmentation performance of *PC* was considered satisfactory by an experienced operator (V.D.). In particular, the final *PC* was trained with approximately 15 annotations per class across 3 TMA cores. Although in this case study we focused on cell-rich and noncell-rich regions, different regions should be annotated for different biological problems, and a novel *PC* should be trained. We provide the source code of the main steps of the QuPath pipeline in the GitHub repository at the following URL: https://github.com/Vsc0/msi-enhanced.

**Figure 1:**
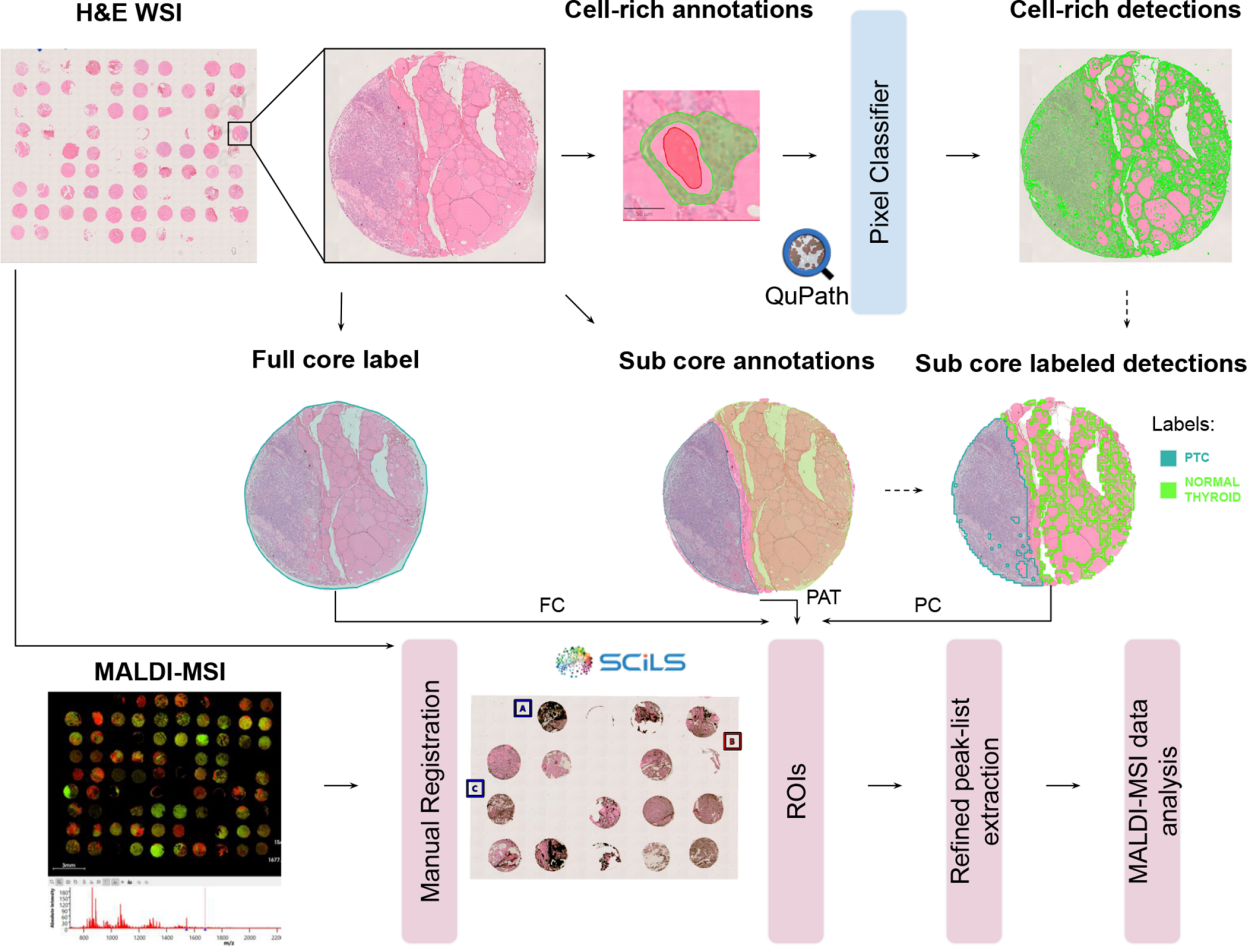
Overview of the proposed workflow to enhance the molecular characterization of thyroid nodules. Cell-rich vs non-cell-rich annotations are manually generated to train the QuPath pixel classifier. The pathologist's subcore annotations and the cell-rich detections are merged (dashed lines) to generate new subcore-labeled detections. In the SCiLS software, the H&E WSI is manually registered with the MALDI-MSI. The labeled ROIs derived from the full core (*FC*) contour, subcore pathologist-guided annotations (*PAT*), and subcore pixel classifier (*PC*) detections are imported, and the relative peak list is extracted for data analysis.

To do so in practice, after the creation of a QuPath project, we first executed the TMA dearrayer. The TMA dearrayer was executed by setting the horizontal and vertical labels for each core, while we used the default values for the remaining parameters. We manually adjusted the TMA grid produced by the TMA dearrayer to improve the alignment with the actual tissue contour of each core. Since not all the tissue regions in the H&E were targeted by the MSI acquisition, we considered valid only the TMA cores whose MSI spot locations were present in SCiLS; an example of H&E with valid cores is reported in Figure S2. For each valid core, we set the resolution parameter to 512 × 512 px, and we extracted patches at 0.44 μm/px from the H&E. The *PC* is a multilayer perceptron—that is, a small artificial neural network—available in QuPath. We selected Gaussian and weighted deviation multiscale features from the Red, Green, Blue, and Hematoxylin image channels. The multiscale features are extracted by applying both a Gaussian and a weighted deviation kernel with the standard deviation parameter set to 2 and 4.^24^ The Gaussian filter extracts general-purpose features like color and pixel intensity, while the weighted deviation feature discriminates between textured and smooth areas. For additional information, we redirect the reader to the official QuPath PC documentation.^21^ The resulting *PC* model architecture is composed of 32 parameters. In the GitHub repository, we provided a Groovy script to load and run the trained *PC*. We executed the *PC* within each TMA core, setting the minimum object size and the minimum hole size parameters to 7 μm^2^. In Figure S3, we show the *PC* predictions on a detail from the original TMA.

We assigned the label of the pathologist subcore annotations to the *PC* detections using QuPath; an example of pathologist subcore annotation is reported in Figure S4. To take into account the large resolution gap between the H&E and MSI *m*/*z* ion images, that is, from 0.2208 to 20 μm/px, we simplified the resulting polygons by slightly expanding and tiling the pixel classifier detections to approximate the 20 × 20 μm/px MSI spot resolution. This polygon simplification step, consisting of a reduction in the number of vertices of the predicted polygons, made the import of the detection in the SCiLS software more efficient. We provided a Groovy script to perform this step in the GitHub repository. In Figure S5, we show the final annotations exported from the QuPath software and imported into the SCiLS software for the MSI data analysis. Notwithstanding the previous example, the provided Groovy scripts (available at https://github.com/Vsc0/msi-enhanced) allow users to transfer pathologist region labels to the *PC* detections and consequently to tile these regions to approximate the MALDI-MSI lateral resolution. This modular approach is adaptable: researchers can retrain *PCs* to distinguish additional classes (e.g., tumor vs stroma) following the official QuPath pixel classifier documentation, thereby tailoring the workflow to their specific biological or clinical questions.

### H&E-Derived Histopathologic ROIs

To perform the comparison of the peak lists (list of *m*/*z* feature intervals) derived from cell-rich regions against those delineated from the full core contours and subcore pathologist annotations, three different types of ROIs were generated in the SCiLS software and then partitioned by diagnosis label. For the first type, each ROI corresponds to the area analyzed with MALDI-MSI in each full TMA core; therefore, the ROI type was named “Full core” (*FC*), as shown in Figure 2A. The other two types were exported from QuPath as SCiLS Exchange Format (*.sef) and imported into the SCiLS dataset. In particular, the ROIs named “*PAT*” correspond to the subcore manual annotations defined by the pathologist, as shown in Figure 2B. The *PC*, cell-rich ROIs were generated using QuPath, as described in the previous section. Contextually, a mean spectrum per ROI type was generated and used to detect the corresponding peak list. Peak-picking was performed in mMass (v5.5.0),^25^ setting the signal-to-noise ratio (S/N) to 6 and the relative intensity threshold to 0.3%. The peak list allows the retrieval of the absolute signal intensity of the *m*/*z* features it contains in each MSI spot within the ROI. We compared the three types of ROI, using the corresponding peak lists, by performing principal component analysis (PCA) and finally receiver operator characteristic (ROC) analyses.

**Figure 2:**
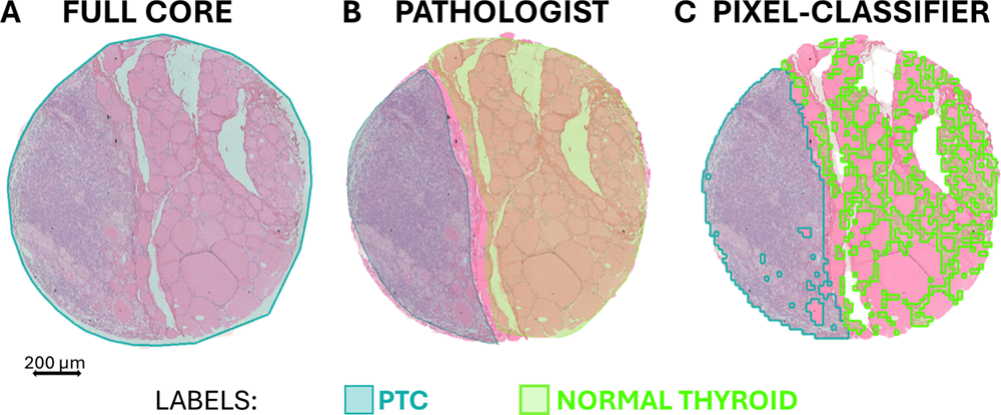
Exemplary figure of the three types of ROIs used. MALDI-MSI regions overlaid on the H&E-stained image of the same TMA core across all panels: (A) full core (*FC*), corresponding to the original MSI measurement area; (B) subcore annotations, manually drawn by the pathologist (*PAT*); (C) ROIs generated in QuPath using the *PC*. A color legend is provided, indicating the label assigned by the pathologist for this core: PTC, teal; normal thyroid, light green.

The *PC* ROIs findings were compared with those obtained from other ROI types (*FC* and *PAT*). PCA analysis was performed in SCiLS, using unit variance for scaling, considering all the individual spectra and setting a maximum of 5 components. ROC analysis was performed by selecting the all-spectra option. An *m*/*z* feature was considered discriminatory for the ROC analysis if the output, that is, the area under the curve (AUC), was greater than 0.7 or less than 0.3.

## Results and Discussion

This study was designed as a *proof of concept* to demonstrate an automated and reproducible workflow for refining pathologist-defined ROIs. The proposed straightforward interdisciplinary approach was used to simplify and improve spatial proteomics data analysis of thyroid nodules acquired by MALDI-MSI.

A dedicated QuPath-trained *PC* was used to specifically select cell-rich ROIs based on the tissue morphology from the H&E staining of the TMA.

### Overall Comparison of the Different ROIs Type

Compared to the *FC*, both *PAT* and *PC* regions had the advantage of including pathology-specific regions (PTC, FVPTC, NIFTP, and FA), excluding stroma and normal thyroid spectra, as shown in Figure 2, where normal thyroid regions are included in the same region as *FC*. The greater specificity of *PC*-derived ROIs can be easily explained by the specific selection of regions containing a high density of cellular nuclei. In this way, cellular protein content was increased, leading to a more precise characterization of the different pathologies included in this study. This particular aspect allows further concentration of pathology-distinct proteomic signatures. However, the manually selected regions (*PAT*) do not comprise dense cellular regions, resulting in an overall signal with additional interfering peaks and, in some cases, signals coming from connective tissue (of the stroma) surrounding or included in the tumor regions. Moreover, *PAT* ROIs strongly suffer from inter-observer variability: different pathologists might annotate different regions, leading to less reproducibility and less precision in the selection of cellular-specific regions. Nonetheless, these annotations can be used to label the regions selected by the *PC*. In fact, a key step to reach the histopathological specificity observed in *PC* ROIs is the labeling inherited by the *PAT* ROIs. An example is reported in Figure 2, where the two labels “PTC” and “normal thyroid” are present in both *PAT* and *PC* ROIs. Here, the important difference lies in the cell-specific selection achieved by *PC*. Indeed, we can appreciate a colloid-rich region in the *PAT* ROI labeled as normal thyroid (in green), while the *PC* ROI specifically follows the cell borders of thyroid follicles, excluding the colloid. Another advantage to note is the automatic selection of these regions, leading to more reproducible results across different specimens analyzed. Finally, the number of pixels examined has significantly decreased as a result of this precise and careful selection of cell-rich areas (FC = 205425 px, PAT = 173027 px, PC = 60929 px). This step further simplifies MSI data elaboration, requiring less computational resources for spatial-omics analysis, and speeding up the molecular characterization of samples with complex histopathological features. The proposed workflow is flexible in terms of the pixel classifier task, the WSI staining (e.g., H&E, Papanicolaou), and the MALDI-MSI modality (e.g., proteomics, lipidomics, glycomics), allowing easy replacement with different or more powerful morphology-specific pixel/instance-classifier predictive models as they become available, given sufficient computational resources for inference.

### Improvement of Signals of Interest

To investigate the informative power of the *PC* ROI, the signal intensity of the contaminant peaks was first explored. The peak lists obtained using mMass (v5.5.0)^25^ were compared with a reference list containing *m*/*z* signals corresponding to the HCCA matrix and trypsin autolysis signals. For the *PC* ROI, around a 15% decrease in the signal intensity of 11 interfering peaks from the HCCA matrix (*m*/*z* 839.08, *m*/*z* 845.09, *m*/*z* 855.05, *m*/*z* 861.06, *m*/*z* 867.08, *m*/*z* 1036.13, *m*/*z* 1044.09, *m*/*z* 1054.08, *m*/*z* 1060.06, *m*/*z* 1066.08) and 3 from trypsin autolysis (*m*/*z* 842.51, *m*/*z* 1045.56, *m*/*z* 1220.64) was observed when comparing it to the *PAT* and *FC* ROIs, as shown in Figure 3A. Conversely, the signal intensity of tryptic peptides significantly increased. In particular, four peptides of interest, previously identified in a comparable MSI dataset,^11^ were investigated in the three ROI types.

**Figure 3:**
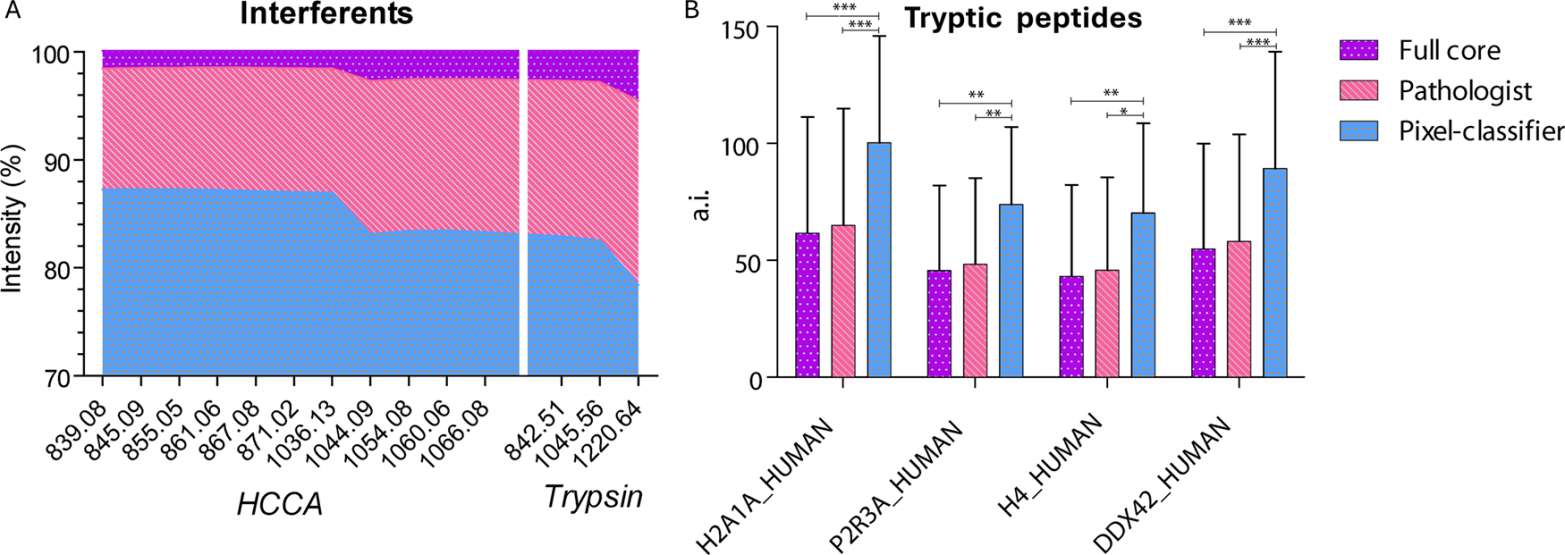
Interferents and peptide signal intensities in full core (*FC*), pathologist (*PAT*), and pixel classifier (*PC*) ROIs. A color legend is provided on the right, illustrating the ROI type and the corresponding color code. (A) Signal intensity of contaminant peaks in the three ROI types. On the *y-*axis, the relative signal intensity of each contaminant *m*/*z* peak on the *x-*axis is provided. The *m*/*z* signals on the left group belong to 4-HCCA peaks, while those on the right correspond to trypsin autolysis peaks. (B) Bar plot reporting the signal intensities of four tryptic peptides: H2A1A_HUMAN, P2R3A_HUMAN, H4_HUMAN, and DDX42_HUMAN (Histone H2A type 1-A, Serine/threonine-protein phosphatase 2A regulatory subunit B” subunit alpha, Histone H4, and ATP-dependent RNA helicase DDX42). The absolute intensity (a.i.) and corresponding error bars in the three ROI types are reported. Two-way ANOVA with Bonferroni post-tests results are reported above the bar plot (*P* < 0.001= ***, *P* < 0.01 = **, *P* < 0.05 = *).

As shown in Figure 3B, the signal intensity of these four analytes showed a significant improvement in *PC* ROIs, compared to both the *FC* (+38%) and the *PAT* (+35%) ROIs. This signal intensity increment is even clearer when looking at the signal intensities of these peptides in different histopathological classes included in the TMA, as shown in Figure S6. Here, we can appreciate a significant signal increase in the malignant conditions (FVPTC and PTC) when comparing not only *FC* and *PC* but also *PAT* and *PC* ROIs. When looking at the NIFTP regions, we can observe that no significant improvement in signal intensity is observed when using manual annotations (*PAT*). However, the *PC* ROIs led to a significant improvement (average +31% compared to *FC* and +30% compared to *PAT*) for peptides of interest intensities. These observations can be extended generally to all peptide signals and lead to more molecularly informative spectra. In fact, when performing peak-picking, the S/N plays a crucial role in feature finding. As a result, a greater number of interfering peaks were found in *FC* spectra, compared to both *PAT* and *PC* regions. Overall, 527 *m*/*z* values were common to the three ROIs' peak lists. Importantly, the *PC* peak list allowed the detection of a greater number of unique (+34% and +95%) and total *m*/*z* values (+9% and +24%) compared to *FC* and *PAT*. Overall, the *PC* ROIs allowed for a decrease of the interfering signals (matrix peaks and trypsin autolysis peaks) while increasing the S/N of the analytes of interest, in this case, tryptic peptides. These results lead to the retrieval of deeper proteomic information from the histopathological classes included in this study.

### Histopathological Discrimination

Since the ultimate scope of the MSI analysis performed on this TMA was to identify possible molecular signatures able to distinguish diverse thyroid pathologies, we compared the ability to discriminate the four pathologic conditions in the three ROI types. To do so, PCA was performed separately on *FC*, *PAT,* and *PC* ROIs. When the PCA obtained from the *FC* ROIs in Figure 4A, a large variability within the four different histopathological regions is observed, and no clear clusters are highlighted. On one hand, with the *PAT* ROIs, a general improvement of the clustering is observed, with a separation of PTC regions from FVPTC, NIFTP, and FA along principal component (PC) 3. On the other hand, FVPTC and NIFTP regions appear to be dispersed in two separate classes each, suggesting that the *PAT* annotations and the relative peak list are not able to define a clear molecular signature for these histopathological regions. Conversely, looking at the PCA of the *PC* ROIs, we can appreciate a separation of the four diagnostic classes according to their aggressiveness along the first component. In fact, a clear cluster for the benign condition (FA) close to the origin of the axes can be observed, while moving along higher values of the PC1, it is possible to see an NIFTP cluster, followed by the FVPTC. The PTC region is separated along the second and third components, suggesting a higher molecular variability compared to the other histologic regions. The NIFTP regions are separated into two clusters along PC2 and PC3. This observation confirms the molecular behavior already observed with MALDI-MSI in a previous work.^11^

**Figure 4:**
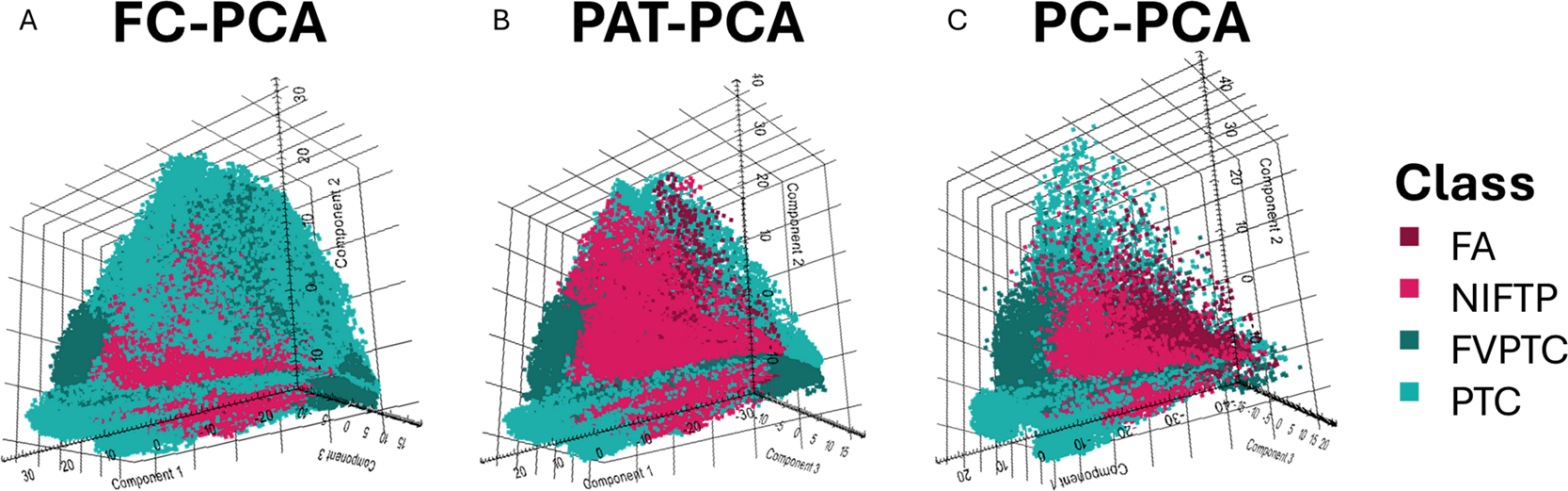
Principal component analysis (PCA) of proteomics profiles obtained from *FC* (A), *PAT* (B), and *PC* (C) ROIs, using the corresponding peak list. 3D PCA score plots showing the clustering of proteomics features extracted from mass spectrometry imaging data. Each point represents a pixel-level spectrum, with the three principal components (Component 1, Component 2, and Component 3) capturing the majority of variance across the datasets. A color legend indicating the corresponding histopathological class is provided on the right.

To further explore the discriminatory capability of the *PC* ROIs compared to *FC* and *PAT*, a ROC analysis was performed using the three peak lists obtained, comparing each diagnostic class to the others. Briefly, the *PC* peak list allowed for finding around 50% more discriminatory *m*/*z* signals compared to the *FC* and *PAT* (*PC* = 41, *PAT* = 21, and *FC* = 22). Nonetheless, when comparing the area under the curve (AUC) for the same *m*/*z* value in the different ROIs, the *PC* data allowed for the highest value in the same comparison, as shown in the example in Figure 5.

**Figure 5:**
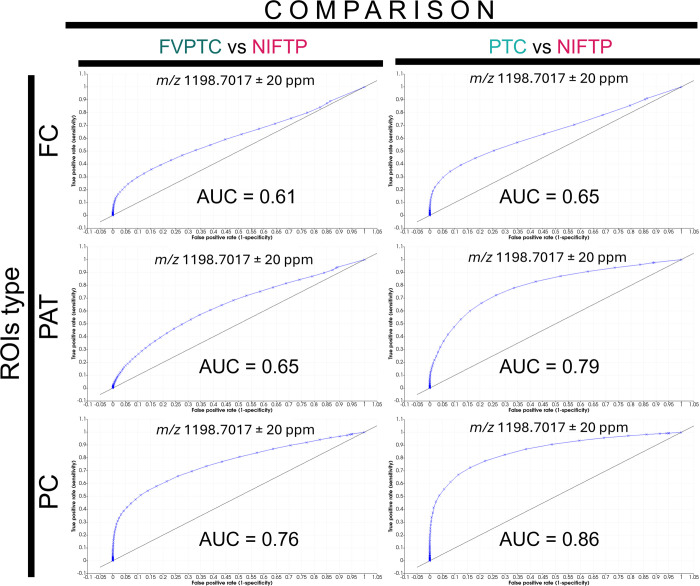
Receiver operating characteristic (ROC) curves for the classification of thyroid tumor subtypes based on the intensity of an exemplary *m*/*z* signal (*m*/*z* 1198.7017 ± 20 ppm). The left column compares FVPTC with NIFTP, while the right column compares PTC with NIFTP, across three types of regions of interest (ROIs): *FC* (top row), *PAT* (middle row), and *PC* (bottom row). The area under the curve (AUC) values indicate classification performance, with higher AUCs reflecting better discrimination. The *PC* ROIs show the highest classification accuracy in both comparisons (PTC vs NIFTP, AUC = 0.86; FVPTC vs NIFTP, AUC = 0.76).

## Conclusions

In this work, we showed how a QuPath PC can be leveraged to enable the precise selection of cell-rich ROIs, effectively minimizing contamination from colloid-rich regions and reducing interfering signals, such as matrix peaks and trypsin autolysis products. Importantly, the refined ROIs retain the original pathologist-assigned labels (e.g., normal, tumor), enabling accurate biological interpretation. The obtained results confirm that the cell-rich ROIs selection achieved with QuPath enhances cell proteomic signatures. As a result, the S/N of tryptic peptides was enhanced, allowing for a deeper proteomic characterization of the histopathological subtypes analyzed. The improved proteomic information is particularly relevant for the discrimination of NIFTP lesions, which present diagnostic challenges due to their histological and molecular overlap with other follicularly patterned thyroid neoplasms. On one hand, by refining molecular characterization, this approach may aid in the accurate classification of NIFTP nodules, reducing diagnostic uncertainty and supporting better clinical decision-making. On the other hand, improving cell-specific molecular signatures might assist the identification of novel putative biomarkers that could be included in routine clinical diagnostic workflows. Finally, the presented work used a single thyroid TMA with multiple diagnostic classes as a proof-of-concept dataset. Future work will employ the *PC* ROI’s selection to multiple thyroid nodules TMAs. By doing so, patient size will be improved to reinforce the statistical strength of novel putative biomarkers for thyroid nodule precise discrimination. This preliminary approach lays the groundwork for further improvement in spatial-omics analysis, in terms of both time and resource efficiency, including computational time and operator-dependent workload.

## Supplementary Material



## Data Availability

Data that support the findings of this study are available at “Repo_pr5c00432,” Bicocca Open Archive Research Data, V1, doi: 10.17632/2phgdzg9 mg.1 (https://board.unimib.it/datasets/2phgdzg9mg).

## Notes

Figure S1. H&E and MALDI-MSI images showing the spatial localization of three different signals in exemplary TMA core with different diagnosis. Figure S2. A detail of the TMA, containing the manually adjusted TMA grid generated with the TMA dearrayer in the QuPath software. Figure S3. A detail of the TMA, containing the pixel classifier predictions within each TMA core. Figure S4. A detail of the TMA, containing the pathologist sub core annotations. Figure S5. A detail of the TMA, containing the final ROIs derived by the pathologist sub core annotations refined by the pixel classifier detections and consequent tiling to approximate the MSI lateral resolution. Figure S6. Barplot reporting the absolute intensity of four tryptic peptides in FC, PAT, and PC ROIs in the four different diagnostic regions. Figure S7. Summary of the proposed workflow to enhance the molecular characterization of thyroid nodules. (PDF)

## Notes

The workflow was implemented in Groovy (executed within QuPath) and Python. The source code is available in the GitHub repository at the following URL: https://github.com/Vsc0/msi-enhanced.

## References

[1] Colley M. E., Esselman A. B., Scott C. F., Spraggins J. M. (2024). High-specificity
imaging mass spectrometry. Annual Review of
Analytical Chemistry.

[2] Baquer G., Sementé L., Mahamdi T., Correig X., Ràfols P., García-Altares M. (2023). What are we imaging? Software tools
and experimental strategies for annotation and identification of small
molecules in mass spectrometry imaging. Mass
Spectrom. Rev..

[3] Piga I., Magni F., Smith A. (2024). The journey towards clinical adoption
of MALDI-MS-based imaging proteomics: from current challenges to future
expectations. FEBS Lett..

[4] Liu H., Pan Y., Xiong C., Han J., Wang X., Chen J., Nie Z. (2022). Matrix-assisted laser
desorption/ionization mass spectrometry imaging
(MALDI MSI) for in situ analysis of endogenous small molecules in
biological samples. TrAC Trends in Analytical
Chemistry.

[5] Kittrell C., Sells B., Young L., Angel P., Drake R. (2025). 471 Defining
proteomic and cellular elements of the pancreatic ductal adenocarcinoma
(PDAC) tumor microenvironment with mass spectrometry imaging. Journal of Clinical and Translational Science.

[6] Kremslehner C., Zoratto S., Sochorova M., Haschemi A., Ponwieser E., Gendronneau G., Marchetti-Deschmann M., Gruber F. (2023). Imaging the skin epilipidome
and the activity of metabolic key enzymes in the senescence process
at single cell level. Free Radical Biol. Med..

[7] Claes B. S., Krestensen K. K., Yagnik G., Grgic A., Kuik C., Lim M. J., Rothschild K. J., Vandenbosch M., Heeren R. M. (2023). MALDI-IHC-guided
in-depth spatial proteomics: targeted
and untargeted MSI combined. Analytical chemistry.

[8] Capitoli G., Piga I., Galimberti S., Leni D., Pincelli A. I., Garancini M., Clerici F., Mahajneh A., Brambilla V., Smith A. (2019). MALDI-MSI as a Complementary diagnostic tool in cytopathology:
a pilot study for the characterization of thyroid nodules. Cancers.

[9] Smith A., Galli M., Piga I., Denti V., Stella M., Chinello C., Fusco N., Leni D., Manzoni M., Roversi G. (2019). Molecular
signatures of medullary thyroid carcinoma
by matrix-assisted laser desorption/ionisation mass spectrometry imaging. Journal of proteomics.

[10] Piga I., L’Imperio V., Capitoli G., Denti V., Smith A., Magni F., Pagni F. (2023). Paving the path toward
multi-omics
approaches in the diagnostic challenges faced in thyroid pathology. Expert Review of Proteomics.

[11] Denti V., Greco A., Alviano A. M., Capitoli G., Monza N., Smith A., Pilla D., Maggioni A., Ivanova M., Venetis K. (2024). Spatially
Resolved Molecular Characterization of Noninvasive
Follicular Thyroid Neoplasms with Papillary-like Nuclear Features
(NIFTPs) Identifies a Distinct Proteomic Signature Associated with
RAS-Mutant Lesions. International Journal of
Molecular Sciences.

[12] Misra S., Dhawan S., Badwal S., Sengupta A., Khosla A., Agarwal S. K., Rao S. (2024). Evaluation
of the follicular patterned
thyroid lesions based on the WHO 2022 criteria with an emphasis on
the grey-zone lesions. Annals of Diagnostic
Pathology.

[13] Nikiforov Y. E., Seethala R. R., Tallini G., Baloch Z. W., Basolo F., Thompson L. D., Barletta J. A., Wenig B. M., Al Ghuzlan A., Kakudo K. (2016). Nomenclature revision for encapsulated follicular variant
of papillary thyroid carcinoma: a paradigm shift to reduce overtreatment
of indolent tumors. JAMA oncology.

[14] L’Imperio V., Coelho V., Cazzaniga G., Papetti D. M., Del Carro F., Capitoli G., Marino M., Ceku J., Fusco N., Ivanova M. (2024). Machine
Learning Streamlines the Morphometric Characterization
and Multiclass Segmentation of Nuclei in Different Follicular Thyroid
Lesions: Everything in a NUTSHELL. Modern Pathology.

[15] Seethala R. R., Baloch Z. W., Barletta J. A., Khanafshar E., Mete O., Sadow P. M., LiVolsi V. A., Nikiforov Y. E., Tallini G., Thompson L. D. (2018). Noninvasive follicular
thyroid neoplasm
with papillary-like nuclear features: a review for pathologists. Modern Pathology.

[16] Gaffney E., Riegman P., Grizzle W., Watson P. (2018). Factors that
drive
the increasing use of FFPE tissue in basic and translational cancer
research. Biotechnic & Histochemistry.

[17] Zhang X.-p., Su D., Cheng Q.-h. (2003). Advantages
and applications of tissue microarray technology
on cancer research. chinese Journal of cancer
research.

[18] Keller B. O., Li L. (2000). Discerning matrix-cluster peaks in matrix-assisted laser desorption/ionization
time-of-flight mass spectra of dilute peptide mixtures. J. Am. Soc. Mass Spectrom..

[19] Keller B. O., Sui J., Young A. B., Whittal R. M. (2008). Interferences
and contaminants encountered
in modern mass spectrometry. Analytica chimica
acta.

[20] Bankhead P., Loughrey M. B., Fernández J. A., Dombrowski Y., McArt D. G., Dunne P. D., McQuaid S., Gray R. T., Murray L. J., Coleman H. G., James J. A., Salto-Tellez M., Hamilton P. W. (2017). QuPath: Open source software for digital pathology
image analysis. Sci. Rep..

[21] Tutorials, Q. Pixel classification. https://qupath.readthedocs.io/en/stable/docs/tutorials/pixel_classification.html#pixel-classification (accessed Sept 23, 2025).

[22] Mofitt R.
A., Marayati R. (2015). Virtual microdissection identifies distinct
tumor- and stroma-specific subtypes of pancreatic ductal adenocarcinoma. Nat. Genet..

[23] Groseclose M. R., Massion P. P., Chaurand P., Caprioli R. M. (2008). High-throughput
proteomic analysis of formalin-fixed paraffin-embedded tissue microarrays
using MALDI imaging mass spectrometry. Proteomics.

[24] Davies, E. R. Machine Vision: Theory, Algorithms, Practicalities; Microelectronics and Signal Processing; Academic Press, 1990.

[25] Strohalm M., Kavan D., Novák P., Volný M., Havlicek V. (2010). mMass 3: a cross-platform software
environment for
precise analysis of mass spectrometric data. Analytical chemistry.

