# Supplementary material for: Improving the Annotation for Spatial Proteomics: A Computational Approach to Enhance Molecular Characterization of Thyroid Nodules
Source: J Proteome Res. 2026 Jan 8;25(2):1152–9. doi: 10.1021/acs.jproteome.5c00432 (PMC12888015; doi:10.1021/acs.jproteome.5c00432)
Supplement: Supplementary file 1 [file pr5c00432_si_001.pdf]

Supplementary Material

Improving the annotation for Spatial Proteomics: A computational approach to enhance molecular characterization of Thyroid Nodules

Vasco Coelho,[†,⊥] Nicole Monza,[‡,⊥] Natalia S. Porto,[‡] Giulia Capitoli,[¶,§] Vincenzo L'Imperio,[∥] Daniele M. Papetti,[†,¶] and Vanna Denti[*,‡]

†*Department of Informatics, Systems and Communication, University of Milano-Bicocca, Milan, IT*

‡*Proteomics and Metabolomics unit, Department of Medicine and Surgery, University of Milano-Bicocca, Monza, IT*

¶*Bicocca Bioinformatics Biostatistics and Bioimaging B4 Center, University of Milano-Bicocca, Monza, Italy*

§*Biostatistics and Clinical Epidemiology, Fondazione IRCCS San Gerardo dei Tintori, Monza, Italy*

∥*Department of Medicine and Surgery, Pathology, University of Milano-Bicocca, Fondazione IRCCS San Gerardo dei Tintori, Monza, Italy*

⊥*These authors contributed equally to this work.*

E-mail: vanna.denti@unimib.it

- **Figure S1** - H&E and MALDI-MSI images showing the spatial localization of three different signals in exemplary TMA core with different diagnosis.
- **Figure S2** - A detail of the TMA, containing the manually adjusted TMA grid generated with the TMA dearrayer in the QuPath software.
- **Figure S3** - A detail of the TMA, containing the pixel classifier predictions within each TMA core.
- **Figure S4** - A detail of the TMA, containing the pathologist sub core annotations.
- **Figure S5** - A detail of the TMA, containing the final ROIs derived by the pathologist sub core annotations refined by the pixel classifier detections and consequent tiling to approximate the MSI lateral resolution.
- **Figure S6** - Barplot reporting the absolute intensity of four tryptic peptides in FC, PAT, and PC ROIs in the four different diagnostic regions.
- **Figure S7** - Summary of the proposed workflow to enhance the molecular characterization of thyroid nodules.

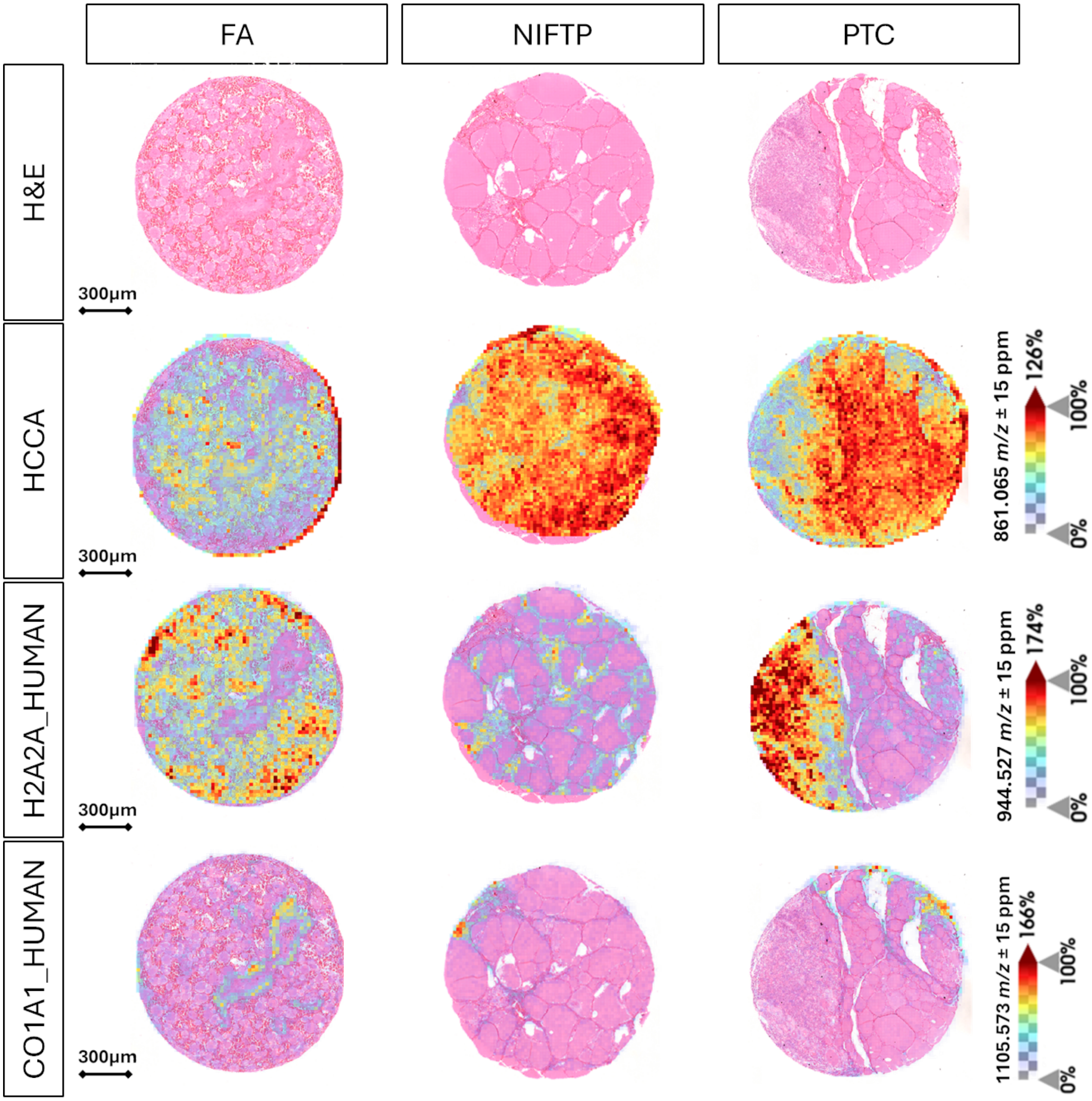

Figure S1: H&E (first row) and MALDI-MSI images (second to fourth row) showing the spatial localization of three different signals in exemplary TMA core with different diagnosis. The interferent signal, HCCA (m/z 861.065 $\pm$ 15ppm), has an aspecific localisation, with higest intensity in correspondence of tissue holes, borders of the TMA or in correspondence of the colloid. In the third and forth row two peptides signals, H2A2A_HUMAN (m/z 944.527 $\pm$ 15ppm) and CO1A1_HUMAN (m/z 1105.573 $\pm$ 15ppm), are shown. For this signals a specific localisation can be observed: the H2A2A_HUMAN peptide shows a specific localisation in correspondence of cell-rich areas, with the highest intensity appreciable in the PTC tumour core. CO1A1_HUMAN is specifically localised in vascular wall and collagen-rich regions. A scale bar on the bottom left is shown, as well as a color-coded scale for signal intensity on the right. H&E, hemathoxylin and eosin; MALDI-MSI, matrix-assisted laser desorption/ionization mass spectrometry imaging; FA, follicular adenoma; NIFTP, noninvasive follicular thyroid neoplasm with papillary-like nuclear features; PTC, papillary thyroid cancer; HCCA, α-Cyano-4-hydroxycinnamic acid; H2A2A_HUMAN, Human Histone H2A type 1-A; CO1A1_HUMAN, Human Collagen alpha-1(1) chain precursor.

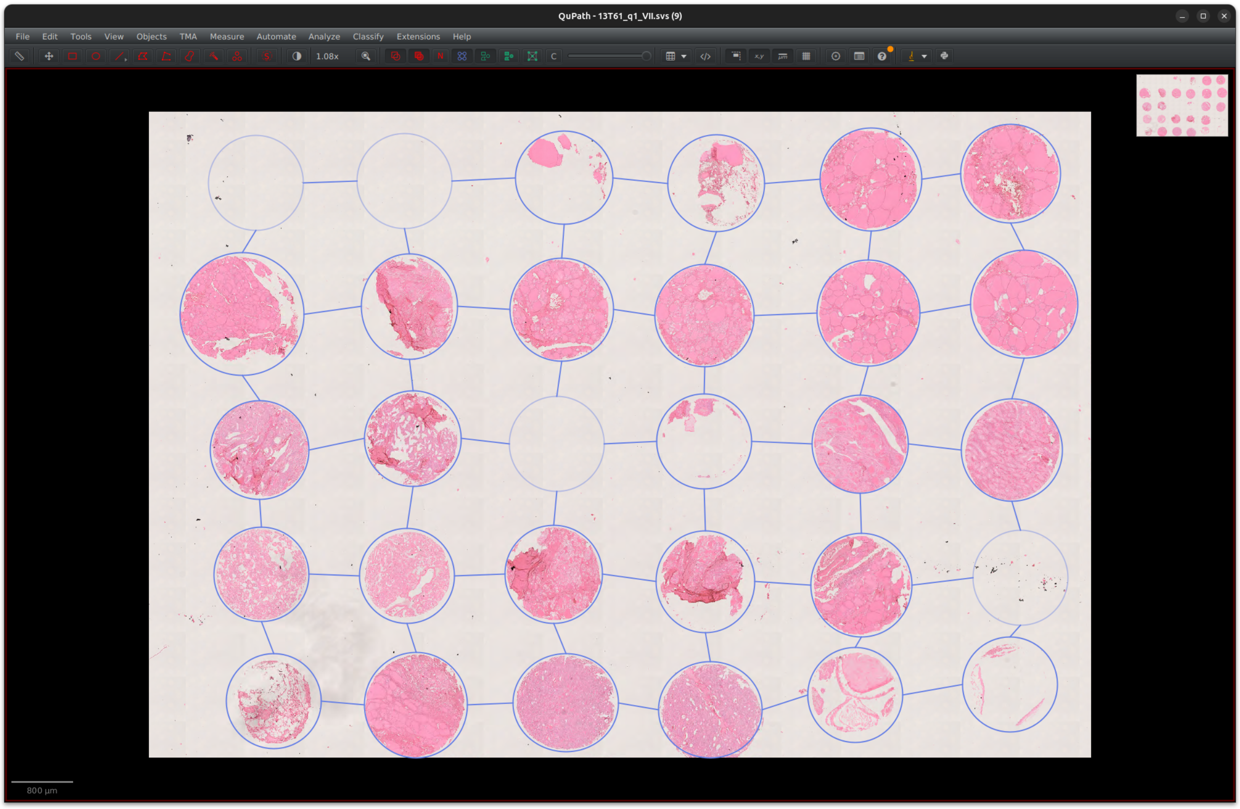

Figure S2: A detail of the TMA, containing the manually adjusted TMA grid generated with the TMA dearrayer in the QuPath software. The valid (dark blue) and missing (light blue) core contours are represented.

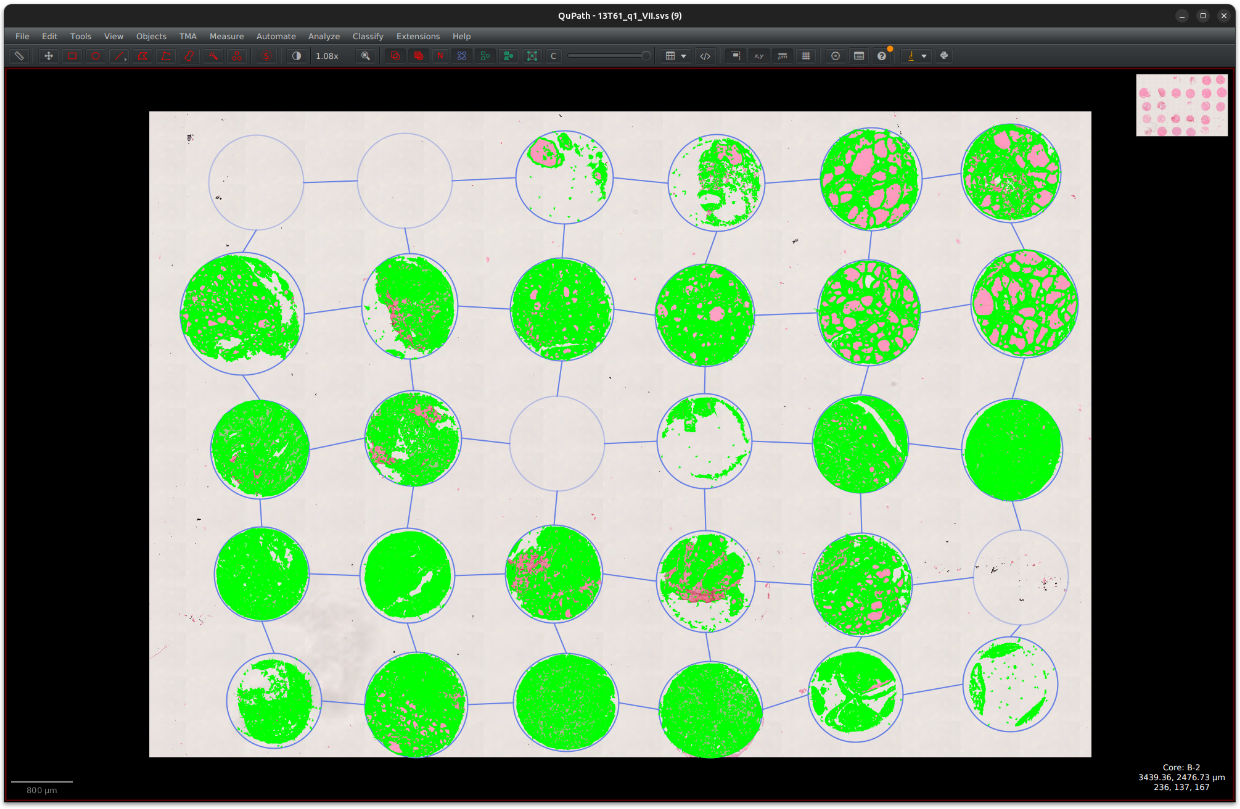

Figure S3: A detail of the TMA, containing the pixel-classifier (PC) predictions within each TMA core. The PC predictions for the cell-rich regions (green) class are shown.

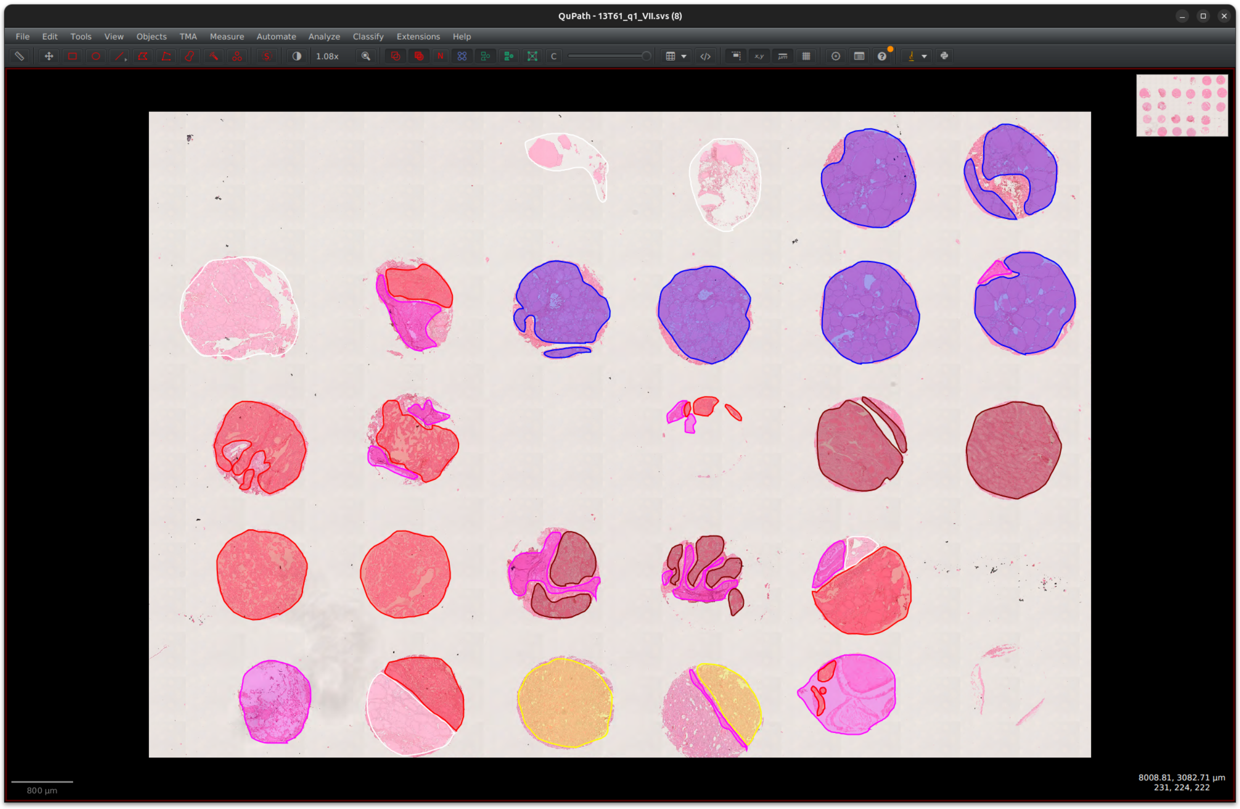

Figure S4: A detail of the TMA, containing the pathologist (*PAT*) sub core annotations. The *PAT* annotations for the PTC (red), FVPTC (maroon), NIFTP (blue), HCA (yellow), S (magenta), and N (white) classes are shown. The FA (cyan) class is not present in this specific detail of the TMA. Only the *PAT* annotations for the PTC, FVPTC, NIFTP, and FA classes were used in the study. PTC, papillary thyroid cancer; FVPTC, follicular-variant of papillary thyroid cancer; NIFTP, noninvasive follicular thyroid neoplasm with papillary-like nuclear features; HCA, Hürthle cell adenoma; S, stroma; N, normal thyroid tissue.

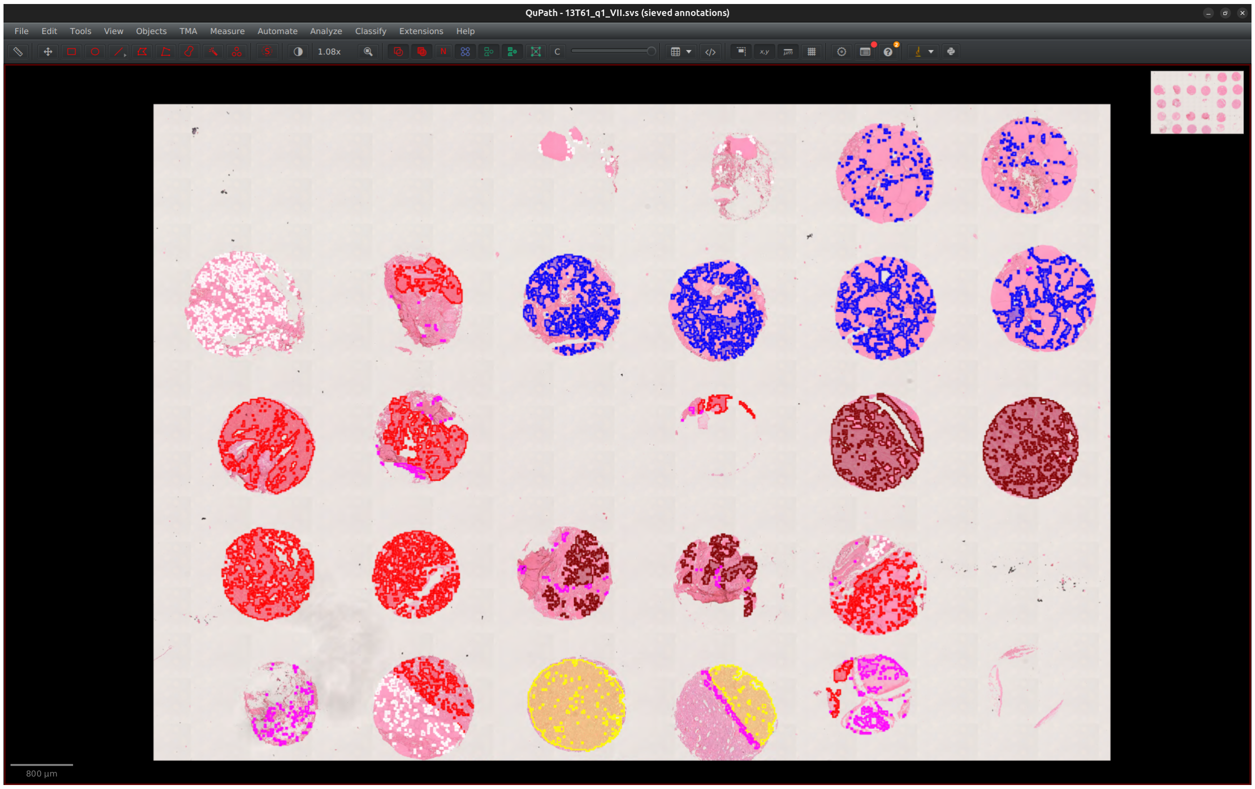

Figure S5: A detail of the TMA, containing the final ROIs derived by the pathologist sub core annotations refined by the pixel-classifier detections and consequent tiling to approximate the MSI lateral resolution. The *PAT* annotations for the PTC (red), FVPTC (maroon), NIFTP (blue), HCA (yellow), S (magenta), and N (white) classes are shown. The FA (cyan) class is not present in this specific detail of the TMA. Only the PAT annotations for the PTC, FVPTC, NIFTP, and FA classes were used in the study. PTC, papillary thyroid cancer; FVPTC, follicular-variant of papillary thyroid cancer; NIFTP, noninvasive follicular thyroid neoplasm with papillary-like nuclear features; HCA, Hürthle cell adenoma; S, stroma; N, normal thyroid tissue.

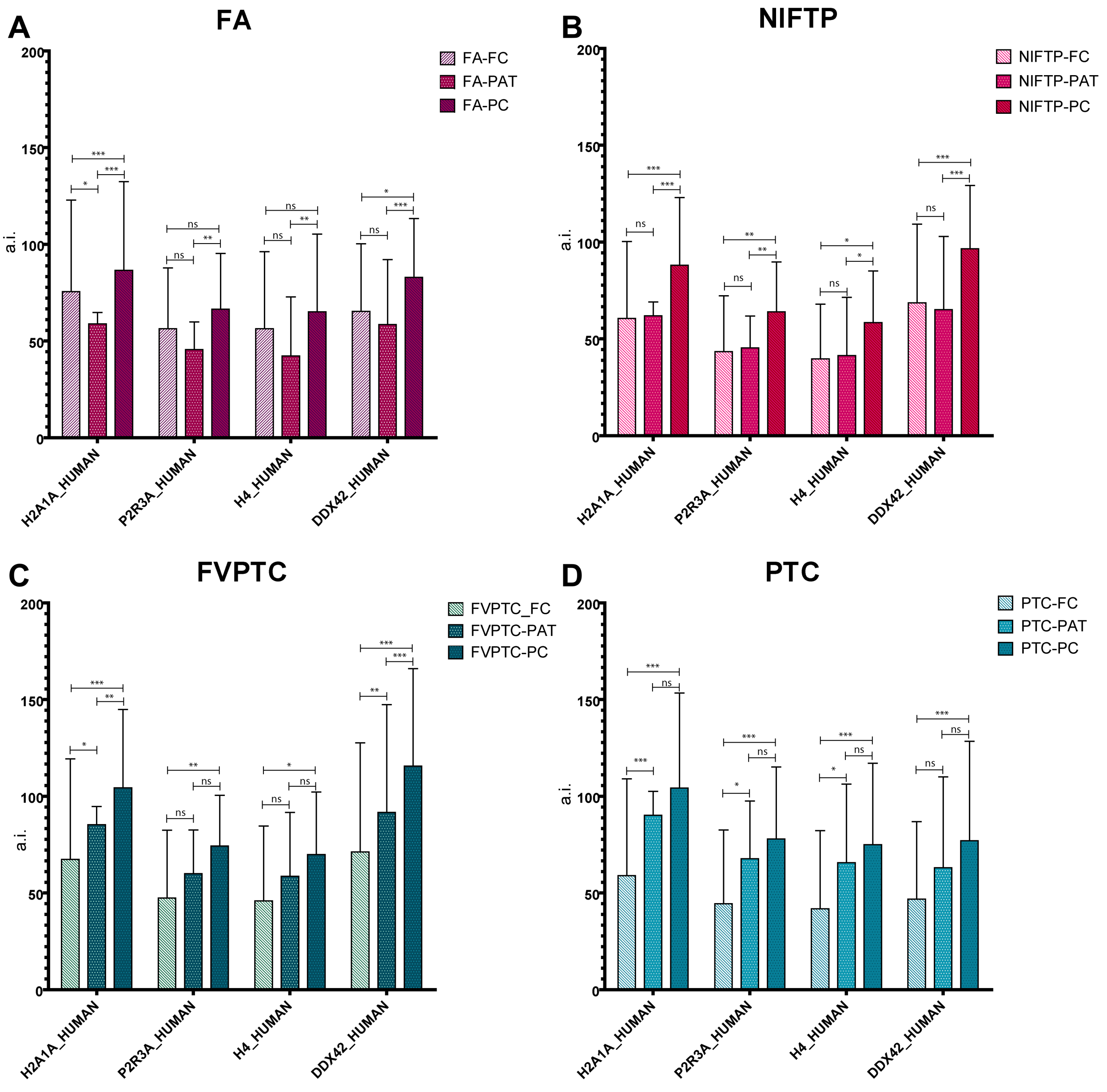

Figure S6: Barplot reporting the absolute intensity (a.i.) of four tryptic peptides (H2A1A_HUMAN, P2R3A_HUMAN, H4_HUMAN and DDX42_HUMAN) in *FC*, *PAT* and *PC* ROIs in the four different diagnostic regions (A. FA, B. NIFTP, C FVPTC, D. PTC). A color legend is provided on the right, illustrating the ROI type and the corresponding color code. The absolute intensity (a.i.) and corresponding error bars in the three ROI types are reported. Two-way ANOVA with Bonferroni post-tests results are reported above the barplot (P<0.001= ***, P<0.01=**, P<0.05=*).

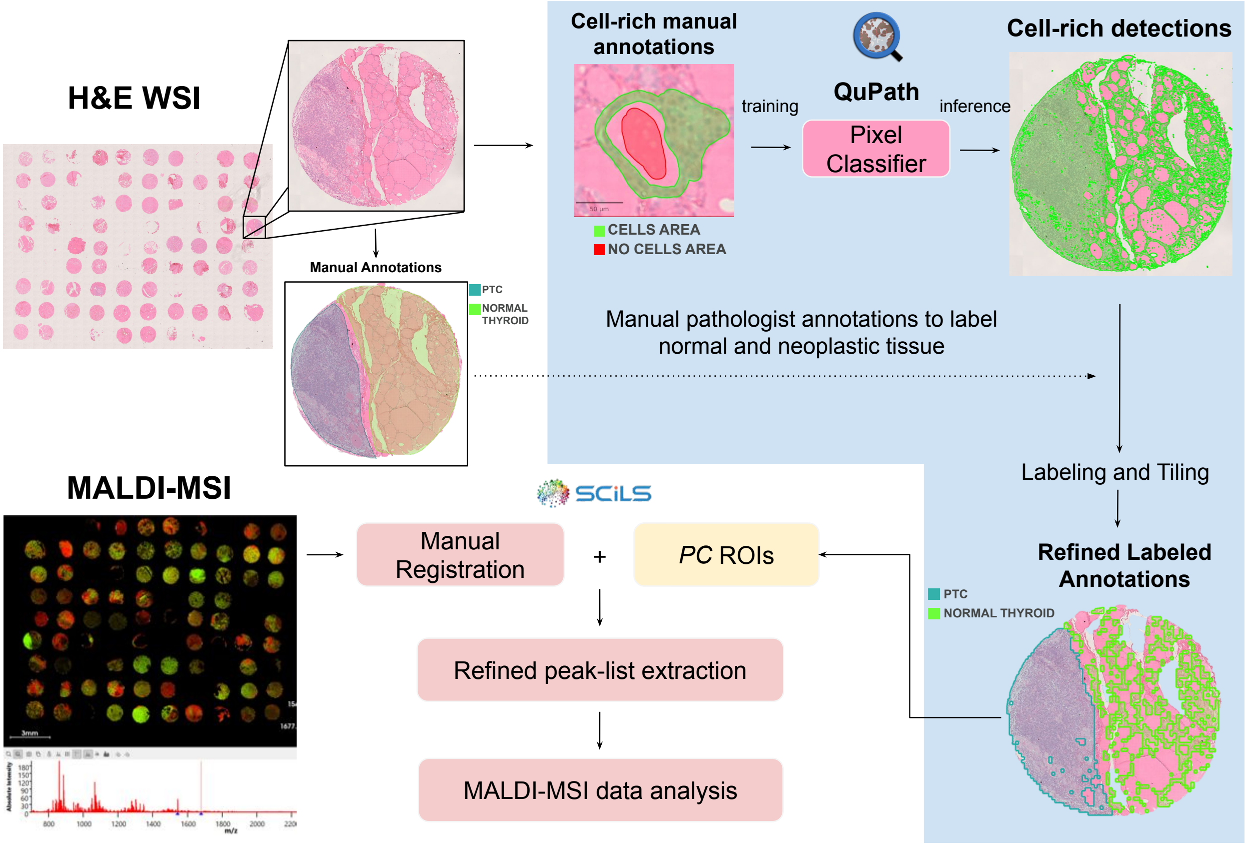

Figure S7: Summary of the proposed workflow to enhance the molecular characterization of thyroid nodules. The steps to identify the cell-rich regions within the pathologist annotations (light blue) are executed within the QuPath software. These regions are then refined into PC-ROIs and subsequently integrated with MSI data to support spatially resolved molecular analysis.
